# Electron Density and Biologically Effective Dose (BED) Radiomics-Based Machine Learning Models to Predict Late Radiation-Induced Subcutaneous Fibrosis

**DOI:** 10.3389/fonc.2020.00490

**Published:** 2020-04-21

**Authors:** Michele Avanzo, Giovanni Pirrone, Lorenzo Vinante, Angela Caroli, Joseph Stancanello, Annalisa Drigo, Samuele Massarut, Mario Mileto, Martina Urbani, Marco Trovo, Issam el Naqa, Antonino De Paoli, Giovanna Sartor

**Affiliations:** ^1^Department of Medical Physics, Centro di Riferimento Oncologico di Aviano (CRO) IRCCS, Aviano, Italy; ^2^Department of Radiation Oncology, Centro di Riferimento Oncologico di Aviano (CRO) IRCCS, Aviano, Italy; ^3^Guerbet SA, Villepinte, France; ^4^Breast Surgery Unit, Centro di Riferimento Oncologico di Aviano (CRO) IRCCS, Aviano, Italy; ^5^Department of Radiology, Centro di Riferimento Oncologico di Aviano (CRO) IRCCS, Aviano, Italy; ^6^Department of Radiation Oncology, Udine General Hospital, Udine, Italy; ^7^Department of Radiation Oncology, University of Michigan, Ann Arbor, MI, United States

**Keywords:** radiomics, radiotherapy, machine learning, breast cancer, fibrosis

## Abstract

**Purpose:** to predict the occurrence of late subcutaneous radiation induced fibrosis (RIF) after partial breast irradiation (PBI) for breast carcinoma by using machine learning (ML) models and radiomic features from 3D Biologically Effective Dose (3D-BED) and Relative Electron Density (3D-RED).

**Methods:** 165 patients underwent external PBI following a hypo-fractionation protocol consisting of 40 Gy/10 fractions, 35 Gy/7 fractions, and 28 Gy/4 fractions, for 73, 60, and 32 patients, respectively. Physicians evaluated toxicity at regular intervals by the Common Terminology Adverse Events (CTAE) version 4.0. RIF was assessed every 3 months after the completion of radiation course and scored prospectively. RIF was experienced by 41 (24.8%) patients after average 5 years of follow up.

The Hounsfield Units (HU) of the CT-images were converted into relative electron density (3D-RED) and Dose maps into Biologically Effective Dose (3D-BED), respectively. Shape, first-order and textural features of 3D-RED and 3D-BED were calculated in the planning target volume (PTV) and breast. Clinical and demographic variables were also considered (954 features in total). Imbalance of the dataset was addressed by data augmentation using ADASYN technique. A subset of non-redundant features that best predict the data was identified by sequential feature selection. Support Vector Machines (SVM), ensemble machine learning (EML) using various aggregation algorithms and Naive Bayes (NB) classifiers were trained on patient dataset to predict RIF occurrence. Models were assessed using sensitivity and specificity of the ML classifiers and the area under the receiver operator characteristic curve (AUC) of the score functions in repeated 5-fold cross validation on the augmented dataset.

**Results:** The SVM model with seven features was preferred for RIF prediction and scored sensitivity 0.83 (95% CI 0.80–0.86), specificity 0.75 (95% CI 0.71–0.77) and AUC of the score function 0.86 (0.85–0.88) on cross-validation. The selected features included cluster shade and Run Length Non-uniformity of breast 3D-BED, kurtosis and cluster shade from PTV 3D-RED, and 10th percentile of PTV 3D-BED.

**Conclusion:** Textures extracted from 3D-BED and 3D-RED in the breast and PTV can predict late RIF and may help better select patient candidates to exclusive PBI.

## Introduction

Subcutaneous radiation induced fibrosis (RIF) is characterized by a progressive induration and thickening of the subcutaneous tissues and is one of the late adverse effects of breast radiotherapy (RT) mostly affecting cosmesis. It is a dose dependent and slowly progressive side effect originating from a proliferative response of surviving fibrocytes to growth factors (e.g., the transforming growth factor β (TGF-β), released in response to tissue injury) (1).

The available tools to predict late subcutaneous fibrosis in patients treated with RT are of limited quality. Models to predict Normal Tissue Complication Probability (NTCP) for RIF after breast RT have been first fitted to published data of rates of incidence from whole breast irradiation (WBI) (2). Later, models for NTCP of RIF have been refined by including dose volume data from simulated dose distributions of WBI (3) and partial breast irradiations (PBI) (4).

Quantitative analysis of medical images could provide information about intensity, shape, size or volume, and texture of tumor or organs at risk that is distinct or complementary to that provided by other data sources (5). Recently, the combination of quantitative analysis of radiological images with Machine Learning (ML) methods, also known as “radiomics,” has been applied also to predict side effects of RT such as lung-injury following Stereotactic Body RT (SBRT) for lung cancer (6), gastrointestinal and genitourinary toxicities (7) and xerostomia (8).

Other 3D information, as dose distribution delivered in RT calculated on pre-treatment Computer Tomography (CT), can be integrated in the radiomics analysis. The textural analysis of dose distribution could provide more detailed spatial information on the 3D dose distribution: it attempts to extract spatial features from dose distribution to predict RT response instead of dose-volume histogram (DVH) typically used in NTCP models. Dosiomics, or integration of dose features from the irradiated lung, has shown to be predictive of radiation pneumonitis with higher accuracy than DVH-based NTCP models (9).

The purpose of the present work is to develop a model to improve the accuracy of prediction of RIF by integrating data from pre-treatment CT, 3D dose distribution and clinical variables. For this purpose, we developed a ML classifier, that is, a predictive model assigning an unseen patient to one of two possible classes: patient with or without RIF during follow-up. Our study is the first, to the best of our knowledge, to derive a classifier for RIF which includes radiomic variables and individual dose data using ML algorithms.

## Methods

### Patient Data

One hundred sixty-five patients treated with breast conservative surgery for an early stage ductal carcinoma who underwent external PBI were retrospectively analyzed. Patient characteristics, with results of univariate statistical tests to investigate correlation with RIF, are shown in Table 1. All patients underwent a complete free breathing pre-treatment planning CT to include all the organs at risk (OAR), according to the RTOG 0413 protocol (10). CTs were acquired with a GE Lightspeed RT (GE Medical Systems, Waukesha, WI) or a Toshiba Aquilion LB (Toshiba Medical Systems Europe, Zoetermeer, the Netherlands) using 120 kVp, 215–300 mAs 5 mm slice thickness, and voxel size ranging from 0.977 to 1.074 mm.

**Table 1:** Patients characteristics with statistical tests to investigate correlation with RIF.

**Categorical variable**	**Patients (%)**	***p-*value (Chi-square test)**
Number of patients	165 (100)	
No RIF	124 (75.2)	
RIF Grade 1	26 (15.7)	
RIF Grade 2	12 (7.3)	
RIF Grade 3	3 (1.8)	
RIF any grade	41 (24.8)	
**Tumor histology**		
Ductal	155 (93.9)	0.540
Lobular	10 (6.1)	
**Laterality**		
Left	73 (44.2)	0.3651
Right	92 (55.8)	
**Quadrant (cm)**		
Upper, outer	80 (48.5.3)	0.056
Upper, inner	32 (19.4)	
Lower, outer	15 (9.1)	
Lower, inter	23 (13.9)	
Central	15 (9.1)	
**Comorbidity**		
No	112 (67.9)	0.658
Yes	53 (32.1)	
**Fractionation regimen**		
40 Gy/10 fx	73 (44.2)	0.5396
35 Gy/7 fx	60 (36.4)	
28 Gy/4 fx	32 (19.4)	
**Chemotherapy**		
No	152 (92.1)	0.064
Yes	13 (7.9)	
**Hormone therapy**		
No	51 (30.9)	0.793
Yes	114 (69.1)	
**Continuous variable**	**Average (95% CI)**	***p*****-value (Wilcoxon test)**
Age (years)	69.8 (61.0–82.9)	0.611
Pathological tumor size (mm)	12.1 (4–25)	0.552
Follow-up (months)	60.2 (17.2–82.9)	0.384

The clinical target volume (CTV) consisted of the lumpectomy cavity, identified by the post-surgery seroma or by the surgical clips, uniformly expanded by 15 mm, limited to 5 mm from the skin surface and 5 mm from the lung-chest wall interface. The planning target volume (PTV) was calculated from the CTV using uniform 3D expansion of 1 cm, then it was limited to exclude the part outside the ipsilateral breast, the first 5 mm of tissue under the skin and the expansion beyond the posterior extent of breast tissue. Breast tissue visible on the pre-treatment planning CT was outlined, according to the RTOG “Breast Cancer Atlas for Radiation therapy planning: consensus definition” (11).

Patients were treated following a hypo-fractionation protocol (12) designed using iso-effective doses for subcutaneous RIF based on NTCP models (4). The hypofractionation schemes consisted of 40 Gy in 10 fractions (73 patients), 35 Gy in 7 fractions (60) and 28 Gy in 4 fractions fractions. The RT technique consisted of “field-in-field” planning (forward-planned intensity modulated RT) (14) using multiple planar and non-coplanar 6-MV photon beams, and delivered by a Trilogy linear accelerator equipped with a kV on-board imager system and a 120-leaves Millennium multi-leaf collimator (Varian Medical Systems, Palo Alto, CA, US).

All treatments were developed using the Eclipse treatment planning system (Varian Medical), and dose calculations were carried out using the anisotropic analytical algorithm (AAA) with a grid resolution of 2.5 mm, taking into account heterogeneity correction. The CT scan, dose matrix and Region Of Interest (ROI) contours were exported in a DICOM format.

Physicians evaluated toxicity at regular intervals by the Common Terminology Criteria for Adverse Events (CTCAE) (version 4.0). Clinical and demographic variables, age, presence of comorbidities (diabetes and rheumatological disorders), tumor histology, laterality and quadrant, administration of chemotherapy and hormone therapy, were considered (954 features in total). The presence of RIF of any grade (grade 1 or more) was assessed every 3 months after the completion of radiation course and scored in a prospective database. Forty-one (24.8%) patients experienced RIF after average 5 years of follow up. Fibrosis of grade 1, 2, and 3 occurred in 26, 12, and 3 patients, respectively. The maximum toxicity score (Grade 4) was not recorded during follow up.

### Radiomic Analysis of BED and RED

Prior to the calculation of radiomic features, resampling to isotropic voxel size was applied to have standardized voxel spacing across the cohort (15). For example, all CT images were resampled to 3 × 3 × 3 mm^3^ (16). Voxel intensities were grouped into 64 equally spaced bins to reduce image noise and normalize intensities across all different patients.

**Figure 1:**
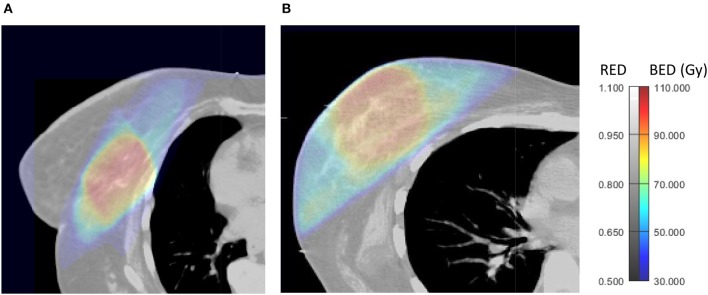
Axial views of 3D-BED and 3D-RED in a patient who did not experience late RIF **(A)** and one who developed late RIF during follow up **(B)**.

In order to remove dependency on the Hounsfield scale used by the two scanners (17), the images were converted from Hounsfield Units to electron density relative to water (3D-RED) using the Hounsfield Units—RED conversion scales of the CTs (Figure 1) as measured on phantom on each CT scanner. Since patients were treated with different fractionation schemes, the 3D dose distributions were converted into 3D Biologically Effective Dose (3D-BED) using the number of fractions of the treatment, and an assumed value of α/β of 3Gy, typical of late-responding tissues as subcutaneous tissue, which has also been used to model RIF (2, 18). A total of 21 shapes, 57 radiomic and 57 dosimetric textural features were calculated from the PTV and breast volume in the 3D-RED and 3D-BED. The radiomic features were calculated following definitions and nomenclature from the Image Biomarker Standardization Initiative (IBSI) (19) using an in-house Matlab code. The in-house code had been previously validated by comparing its results with the Ibex open source software (20). The same features were also calculated after application of one between Gaussian, Laplacian of Gaussian (LoG), or Median filtering to 3D-RED and 3D-BED. The clinical variables follow up, age, tumor location, pathological tumor size, chemo and hormone therapy, were also collected and included in the analysis, so that the variables were 954 in total. A common problem in application of ML classifiers is that some classes have a significantly higher number of examples, a problem which is referred to as class imbalance. The effect of imbalanced datasets on ML performance is detrimental (21, 22), and there are two methods for overcoming this issue, namely under-sampling and over-sampling, of which the latter has been proven to be more effective in ML (21).

We then applied the Adaptive Synthetic Sampling Method for Imbalanced Data (ADASYN) over-sampling technique (23), an improved variant of the Synthetic Minority Over-sampling Technique (SMOTE), which generates synthetic data points by interpolating new feature values between the minority instance and its neighbors, according to the Euclidean distance, in the feature space. In ADASYN, the new minority samples are generated using a density distribution based on the number of out-of-class neighbors so that a minority instance surrounded by more out-of-class instances is considered hard-to-train, and is thus given a higher probability to be augmented (24).

ADASYN was applied to the level that the imbalance was completely eliminated, resulting in an augmented dataset of 252 patients, of which 50% had late RIF. All the analysis was performed using Matlab (Mathworks, Natick, MA).

### ML Models

The occurrence of RIF in patients was converted into a binary outcome, positive for patients who experienced any grade (one or more) of RIF, and negative for patients who did not experience RIF during follow up.

The ML process includes two phases (5). First, to prevent overfitting, prior to applying ML classification, Stepwise forward feature selection was used to select a subset of variables best suited to predict late fibrosis. In Stepwise feature selection, terms from a generalized linear model are removed or added in order to find the subset of variables in the data set resulting in the smallest model with lowest prediction error (25).

Forward stepwise selection is a wrapper method of feature selection, that is, a method which uses a learning technique, in our case a generalized linear model (GLM), to evaluate the importance of the features. Forward selection starts with an empty model. Then at each iteration, the single feature that best improves the fit of the GLM according to a specified criterion is determined (26). As a criterion we used the deviance of the values predicted by the GLM from the test data in a 5-fold cross validation. This is repeated until a best subset predictors (features) are selected.

In order to choose a proper number of variables, the process was initially performed with 4 variables allowed in the feature selection, then repeated with increasing number of variables.

After feature selection, the following binary ML classifiers were applied to the dataset to predict RIF:

1. SVM, which, by means of a kernel function, projects the data into a higher-dimensional feature space and determine a hyperplane in this feature space which separates data points into two categories (27). During the optimization, the proper box constraint level and kernel scale are chosen.
2. Ensemble machine learning (EML) which aggregates multiple learners into a single learner. Decision Trees were used as weak learners (5). During training, the best ensemble EML algorithm is selected between Random forests, Adaptive Logistic Regression and various boosting algorithms: Adaptive, Gentle, and Random Undersampling boosting (28) as well as the optimal number of learning cycles, learning rate, and minimum leaf size.
3. Naïve-Bayesian (NB) classifier which calculates the probability of each class assuming the conditional independence of the attributes using the Naive Bayes formula. A new instance is classified into the class with maximum calculated probability (29, 30). The optimizer also searches the best type of probability distribution (Gaussian or Kernel) and width of the kernel function.

The model to predict the occurrence of RIF was chosen according to the following criteria. First, the performance of the models was evaluated by calculating the average and 95% confidence intervals of sensitivity and specificity of the classifier and the AUC of the score function used by the classifiers in a 5-fold cross validation repeated 500 times in the augmented dataset. Finally, the sensitivity, specificity, and AUC were recalculated on the original, non-augmented dataset.

The models were required to have at least sensitivity and specificity of 0.75, and AUC of the model score of 0.85. Second, models were required to provide a realistic description of occurrence of RIF vs. BED variables. For this purpose, the score used by the best performing models to predict RIF was calculated vs. variables from 3D-BED variables. For biological consistency, models were required to have a continuously monotonic response to increasing dose. Models with non-monotonically increasing dose response were discarded, as this would imply that two different doses can lead to the same risk of side effects and that increasing dose could reduce the risk (31).

## Results

The variables selected for ML are shown in Table 2. Two were textural variables of 3D-BED from the breast, cluster shade and Run Length Non-uniformity (RLN) after application of LoG filter, two were histogram (kurtosis and range) and one textural (Gray Level Co-occurrence Matrix Cluster shade) features (19) from the 3D-RED in the PTV and two histogram (10th percentile and inverse variance) variables of 3D-BED in the PTV. Among these, three variables (RLN of 3D-BED in breast, kurtosis of 3D-RED in PTV, 10th percentile of 3D-RED in PTV) were significantly correlated with occurrence of RIF according to the Wilcoxon-Mann-Whitney test for independent samples. No clinical variable was selected in the model.

**Table 2:** Features selected to predict late fibrosis.

**Image (3D-RED/ 3D-BED)**	**Filter**	**ROI**	**Variables**	**Wilcoxon-Mann–Whitney test *p***
3D-BED	LoG	Breast	Cluster shade	0.1389
3D-BED	LoG	Breast	RLN	0.0084
3D-RED	None	PTV	Kurtosis	0.0238
3D-RED	Gaussian	PTV	Range	0.1021
3D-RED	Gaussian	PTV	Cluster shade	0.6687
3D-BED	Gaussian	PTV	10th Percentile	0.0054
3D-BED	LoG	PTV	Variance	0.1624

*LoG, Laplacian of Gaussian filter; RLN, run length non-uniformity*.

EML with Adaptive Boosting was the best performing model for any number of variables, and it scored an AUC of the radiomic signature of 0.87 (0.85–0.90) with only 6 variables. SVM was the second best performing classifier as it achieved acceptable scores with 7 variables, while Native Bayes gave generally poor performance in terms of specificity (Table 3).

**Table 3:** Performances of different models as a function of increasing number of variables allowed.

**Model**	**Number of variables**	**Cross-validation in the augmented dataset, with 95% CI**			**Original (non-augmented) dataset**		
		**Sensitivity**	**Specificity**	**AUC**	**Sensitivity**	**Specificity**	**AUC**
SVM	4	0.77 (0.74–0.80)	0.69 (0.66–0.71)	0.80 (0.79–0.81)	0.68	0.70	0.78
	5	0.82 (0.79–0.84)	0.68 (0.65–0.71)	0.83 (0.82–0.84)	0.73	0.66	0.81
	6	0.85 (0.83–0.87)	0.71 (0.68–0.73)	0.85 (0.84–0.86)	0.81	0.73	0.84
	7	0.83 (0.80–0.86)	0.75 (0.71–0.77)	0.86 (0.85–0.88)	0.81	0.77	0.86
	8	0.84 (0.81- 0.87)	0.76 (0.73–0.78)	0.88 (0.87–0.88)	0.83	0.81	0.89
EML	4	0.78 (0.73–0.84	0.73 (0.68–0.78)	0.83 (0.80–0.85)	1.00	1.00	1.00
	5	0.84 (0.79–0.88)	0.73 (0.69–0.78)	0.87 (0.84–0.90)	1.00	1.00	1.00
	6	0.86 (0.81–0.89)	0.77 (0.73–0.82)	0.87 (0.85–0.90)	1.00	1.00	1.00
	7	0.87 (0.82–0.91)	0.78 (0.73–0.84)	0.91 (0.88–0.93)	1.00	1.00	1.00
	8	0.89 (0.84–0.94)	0.78 (0.73–0.81)	0.92 (0.90–0.94)	1.00	1.00	1.00
NB	4	0.88 (0.84–0.91)	0.44 (0.41–0.47)	0.65 (0.63–0.68)	0.90	0.46	0.71
	5	0.92 (0.90–0.93)	0.44 (0.42–0.47)	0.82 (0.81–0.83)	0.90	0.45	0.71
	6	0.91 (0.88–0.92)	0.47 (0.45–0.49)	0.82 (0.81–0.83)	0.90	0.46	0.71
	7	0.89 (0.86–0.91)	0.40 (0.35–0.43)	0.78 (0.76–0.81)	0.90	0.45	0.71
	8	0.95 (0.94–0.95)	0.36 (0.34–0.38)	0.80 (0.78–0.82)	0.90	0.45	0.71

*For each model and number of variables, the specificity and sensitivity of the classifier and the AUC with 95% CI calculated in repeated cross-validation are reported, as well as the specificity, sensitivity and AUC in the original (non-augmented) dataset*.

To interpret the features, their values were investigated in the two subsets of patients having extreme values of the function score. These patients were chosen as the 5% with the lowest score function among those without RIF, and the 5% with the highest score function of those who had RIF. Their features are shown in Table 4.

**Table 4:** Values of radiomic variables of the patients with low **(A)** and high **(B)** risk of RIF.

**Image:**	**3D-BED**	**3D-BED**	**3D-RED**	**3D-RED**	**3D-BED**	**3D-BED**	**3D-BED**
Filter:	Log	LoG	None	Gaussian	Gaussian	Gaussian	LoG
ROI:	Breast	Breast	PTV	PTV	PTV	PTV	PTV
Feature:	Cluster shade	RLN	Kurtosis	Range	Cluster shade	Percentile area 10	Variance
**(A)**							
Patient:							
1	−22517	0.58	441.8	3.6	15.2	91.0	0.31
2	−8696.7	0.55	50.7	0.47	−2250.1	74.8	0.43
3	−4993.3	0.62	320.5	1.64	410.6	87.3	0.42
4	−32445.8	0.59	64.2	0.66	−714.9	80.4	0.38
5	−17231.7	0.60	176.7	1.83	400.1	88.4	0.43
6	−10022.4	0.47	15.7	0.90	−2546.9	65.6	0.41
7	35401.4	0.63	92.4	0.64	131.1	90.4	0.39
8	−5332.1	0.54	19.8	0.52	−9583.5	52.41	0.41
Average:	−8229.7	0.57	147.7	1.28	−1767.3	78.8	0.40
**(B)**							
Patient:							
1	−18557.7	0.62	19.9	0.75	−3129.0	82.2	0.44
2	−3426.54	0.60	23.4	0.71	−2300.7	84.7	0.48
3	−53664.9	0.70	13.5	0.65	−3202.5	82.3	0.43
4	−80106.7	0.51	1.9	0.19	539.1	94.0	0.47
5	−29002.7	0.57	15.9	0.56	−6021.6	84.8	0.44
6	−29432.7	0.58	18.1	0.70	−1762.5	81.0	0.46
7	−10230.9	0.63	17.1	0.71	−2367.8	88.7	0.47
Average	−32060.3	0.60	15.7	0.61	−2606.4	85.4	0.46

*These were defined as the 5% patients without RIF and with the lowest function score and the 5% patients with RIF with the highest function score, respectively*.

**Figure 2:**
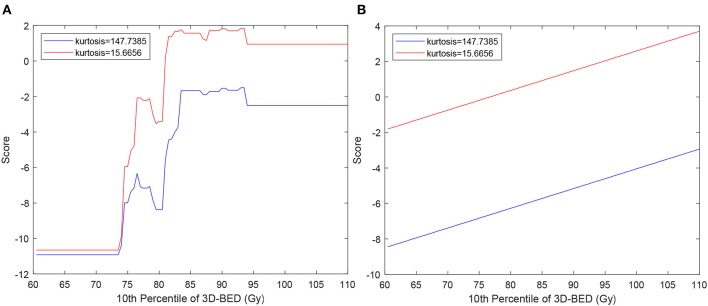
Score function of EML **(A)** and SVM **(B)** used to classify patients vs. 10th percentile of 3D-BED in the PTV. The curves are calculated for values of kurtosis typical of patients at low and high risk of RIF, chosen as average of kurtosis in the 10 patients without RIF with lowest score (blue lines) and in 10 patients with RIF with highest score (red lines).

The score functions of the SVM and EML classifiers, were plotted against the 10th percentile of 3D-BED in the PTV for two values of kurtosis, that is, the average values of the patients at low and high risk of RIF, with the other features fixed at their average values among all the patients (Figure 2). The EML model was then discarded, as it showed a non-monotonically increasing dose-score function. The 7 variables SVM, scoring sensitivity 0.83 (95% CI 0.80–0.86), specificity 0.75 (95% CI 0.71–0.77) and AUC of the score function 0.86 (0.85–0.88) on cross-validation, was chosen as the preferred model. The model had sensitivity, specificity and AUC of 0.81, 0.77, and 0.86 respectively in the original dataset.

## Discussion

Supervised ML methods have been increasingly used in medicine, especially in the field of radiomics (32) to identify patients as responders or not responders but also to predict side effects in OARs (13, 13, 27). They are prone to overfitting, an event in which the model will better reflect noise in the image than the data themselves (33). Hence, careful feature selection and validation must be performed to tackle this limitation. In our results, EML is an example of ML models which overfitted the data, as it provided the best performance on repeated cross-validation for any number of variables, but produced unrealistic dose-response (Figure 2A) which was not monotonic. It was then discarded in favor of the SVM, whose score was monotonically increasing as a function of dose (Figure 2B). SVM models are more robust to overfitting than other ML methods such as decision trees (34), because they tolerate some points on the wrong side of the hyperplane, thus improving model robustness and generalization (5). Because the AUC of the SVM model of 0.86 on the non-augmented dataset is considered excellent (35) and, as the sum of sensitivity and specificity is 1.58, larger than 1.5, the model fulfills the rule of thumb for being useful of a clinical test (36), it can be used to stratify patients according to the risk of subcutaneous RIF.

To the best of our knowledge, this is the first study using radiomic features extracted from dose distribution after conversion to 3D-BED, which was necessary since our patients had different fractionation schemes. As the BED variables were the most correlated with RIF, our analysis confirms that radiation-induced RIF is governed by BED calculated with α/β = 3 Gy to the whole breast and to the high dose region, the PTV. This result is in agreement with previous clinical findings showing that fibrosis is related to dose and dose per fraction (18). On the other hand, a correlation between RIF and maximum dose has been observed in clinical data for both WBI (37) and PBI (38). RIF after PBI has been related to minimum PTV dose (39).

The features that were most correlated with RIF were BED features10th percentile and variance in the PTV and RLN of the breast, cluster shade to both PTV and breast.

Among the BED features, the 10th percentile in PTV is a descriptor of the minimum BED to the PTV, and describes the dependency of fibrosis on the lowest fractionation-corrected dose covering at least 90% of PTV. Cluster shade of BED in the PTV describes asymmetry of the GLCM. A larger module of cluster shade implies large GLCM asymmetry (19), which means that there are regions in the PTV with large differences in BED from their neighbors and may be related to the presence of hot spots in the PTV. Of note, if a PTV is close to the patient's surface, like in Figure 1B, there is a sudden change of dose in the build-up region which may increase cluster shade of BED. These associations are confirmed by larger variance of BED to the PTV in patients with RIF (Table 4).

RLN of BED in the breast describes the similarity among run lengths, defined as the lengths of consecutive voxels having the same dose value in a specified direction, in number of voxels (19) throughout the breast. RLN is related to homogeneity of dose, and lower values indicate more homogeneity among run lengths in the image. In our results patients without RIF had lower RLN of BED (more inhomogeneity). This may be due to larger “out-of-field” areas of the breast in patients less at risk of fibrosis (Figure 1A) that, being irradiated with low, uniform doses from scattered radiation, tend to have larger runs of voxels with the same values of dose from scattered radiation. An example of this situation can be observed in Figure 1A, and suggests that a steep dose gradient outside of the PTV may be beneficial to prevent fibrosis.

These findings indicate that the radiomic BED variables show that higher BED and presence of hot spots of BED in the PTV, as well as higher volumes receiving intermediate doses out of the PTV, as in Figure 1B, are related to occurrence of fibrosis.

The hypothesis underlying the application of radiomics to predict side effects in OARs is that a patient who is more at risk of side effect has a particular appearance of the organ at risk in pretreatment CT from the patient at lower risk. Often, these models are still perceived as “black boxes,” meaning that it is difficult to determine how they arrive at their predictions, which impairs their use by clinicians as part of their clinical practice (40, 41). To address this issue, we provide interpretation of the radiomic features that are selected by the models. In our results, it was found that 3D-RED kurtosis in the PTV was correlated with a higher risk for RIF. Because kurtosis describes inhomogeneity of the electron density of the breast, the patients with more inhomogeneous breast (small kurtosis) are more sensitive, that is, have higher function score for all dose values (Figure 2). Fat, which is radiolucent, appears dark on a CT, while epithelial and stromal tissue appear radiodense and may represent connective tissues (42, 43). Senescence may in the human mammary epithelium be at the origin of RIF (44) and RT may have a more pronounced effect on stroma (42). Thus, an already dense breast could be more prone to developing fibrosis. As younger patients have more inhomogeneous breast, this result seems in agreement with studies reporting worse cosmetic results in young patients [e.g., (45)], who typically have a denser breast. In our results, however, fibrosis was not correlated with age, neither kurtosis (Pearson correlation *p* = 0.28). This lack of correlations with age could be due to the limited range of age of our patients (95%CI 61.0–82.9 years), that do not include younger (<50) patients. The relationship of fibrosis and radiomic features from CT of the breast with age therefore could be the subject of future investigation.

## Conclusion

The models implemented show that radiomic and dose textural variables extracted from the breast and PTV volumes after correction for fractionation and CT density scale can predict RIF and may help better select patients candidate to exclusive PBI.

## Data Availability Statement

The datasets generated for this study are available on request to the corresponding author.

## Ethics Statement

The studies involving human participants were reviewed and approved by Comitato Etico Unico Regionale—CEUR Friuli Venezia Giulia. Azienda Regionale di Coordinamento per la Salute (ARCS), via Pozzuolo n. 330 – 33100 Udine (palazzina B). The patients/participants provided their written informed consent to participate in this study.

## Author Contributions

MA and LV conceived the study. LV and AC performed the contouring of the cases and were responsible for the clinical evaluation of side effects during the follow-up. GP and ADr extracted the imaging and dose data. GP processed the data and performed radiomics analysis. MA performed the machine learning and statistical analysis and wrote the manuscript with inputs from JS, IN, MT, ADP, MU, SM, MM, and GS. All the authors discussed the results and contributed to final manuscript.

## Conflict of Interest

JS is employed by the company Guerbet SA, Villepinte, France. The remaining authors declare that the research was conducted in the absence of any commercial or financial relationships that could be construed as a potential conflict of interest. The handling editor declared a past collaboration with two of the authors MA and IN.
